# American Veterinary College

**Published:** 1895-04

**Authors:** 


### AMERICAN VETERINARY COLLEGE.

The twentieth Annual Commencement exercises of the American Veterinary College, were held at Chickering Hall, Monday evening, March 25, 1895. The programme was opened by the rendition of several selections by the 7th Regiment orchestra. Prayer was offered by Rev. Dr. Daniel G. Wylie. The list of successful graduates was read by Professor W. J. Coates, after which the degree of D.V.S. was conferred by Dr. Fanueil D. Weisse, President of the Board of Trustees, upon the following gentlemen: Augustus Hall Blake, Richmond Hill, N.Y.; Homer Harris Butler, New York, N. Y.; Harry Baker Cox, Merchantville, N. J.; Joseph Francis Devine, New York, N. Y.; Martin Joseph Dair, New York, N. Y.; George B. Gillmor, Pittsburg, Pa.; Robert Johnston Halliday, Jersey City, N. J.; Frank Reed Hanson, New York, N. Y.; Edwin Allen Hogan, Bayonne, N. J.; Moses Isaac, New Haven, Connecticut; Nicholas John Kaiser, Newark, New Jersey, James Michael Laffan, Irvington, N.Y.; William Henry Lawes, Jr., Shrewsbury, N. J.; Aquila Mitchell, Skaneateles, N. Y.; John Joseph Monyhan, Holyoke, Mass.; Francis Theobald O'Sullivan, New York, N. Y.; Bryer Hamilton Pendry, Brooklyn, N. Y.; Clarence R. Shults, Ogdensburg, N.Y.; Aaron Silkman, Goldensbridge, N. Y.; Wright J. Smith, Kingston, N. Y.; and John Newkirk Wrong, Sedalia, Missouri.

Professor L. H. Friedburg awarded the following prizes: The gold medal for the best examination to Aquila Mitchell. The alumni prize, consisting of a set of books, to Frank Reed Hanson, who also received the prize for the best practical examination, and also won the senior anatomy prize. The College Association prize for the best paper read and defended before that organization, consisting of a set of instruments, was won by M. J. Dair. The free scholarship prize for the second-year class, requiring a general average above 75 per cent. was won by Samuel S. Buckley. The half-scholarship prize for the best examination by a first year student was won by Jas. B. Laffan, who also won the junior anatomy prize.

The valedictory address was delivered by Bryer Hamilton Pendry. The address to the graduates was by Dr. Wm. J. O'Sullivan, L.L.D., M.R.C.V.S., of New York City.

The college exercises were completed by a banquet at "Clark's," West Twenty-third Street, where, at 11 P.M., about sixty members of the Alumni Association gathered around the festive board and enjoyed the generous spread provided. The following toasts were responded to: "The Board of Trustees" by Dr. F. D. Weisse; "The Ladies" by Professor C. A. Doremus; "Few Words" by Professor Jas. L. Robertson; "The Alumni Association" by Dr. H. D. Hanson; "Remarks" by Professor L. H. Friedburg; "Colleges Abroad" by Dr. Thomas Giffen; "The Advance of Veterinary Science" by R. S. Huidekoper, M.D. Other toasts were responded to by various members of the different classes. Toast Master, Professor J. E. Ryder.

The classes of '79, '80, '81, '83, '84, '86, '87, '88, '89, '90, '91 '92, '93, '94, and '95 were represented at the alumni meeting and around the table, making the occasion one of the most enjoyable that the graduates of the American Veterinary College have enjoyed for many years.

